# Nutrient Rich Foods Index NRF9.3 Rates Nutrient Density of Kiwifruit in the US and in France

**DOI:** 10.3390/foods15183232

**Published:** 2026-09-12

**Authors:** Adam Drewnowski, Faraja Yusto Mgaya

**Affiliations:** 1 Center for Public Health Nutrition, University of Washington, Seattle, WA 98195, USA; 2 Department of Global Health, University of Washington, Seattle, WA 98195, USA

**Keywords:** Nutrient-Rich Food Index NRF9.3, Food and Nutrient Database for Dietary Studies (FNDDS), ANSES/CIQUAL database, kiwifruit, nutrient density, vitamin C, potassium

## Abstract

This study used the Nutrient-Rich Food (NRF9.3) index to compare the overall nutrient density of kiwifruit with that of other fruits and juices in two national nutrient composition datasets: the US Food and Nutrient Database for Dietary Studies (FNDDS) and the French ANSES/CIQUAL database. Additional data for five kiwifruit cultivars came from the New Zealand Food Composition Tables (NZFCT). NRF9.3 scores were calculated for kiwifruit, citrus fruit, berries, other fruit and juice, cooked and dried fruit, exotic fruit, and fruit cocktails and salads. The NRF9.3 components were the positive NR9 subscore that included protein, fiber, calcium, iron, potassium, magnesium, and vitamins A, C, and E, and a negative LIM that was based on saturated fat, added sugar, and sodium. Kiwifruit (n-6) had the highest mean NRF9.3 scores in both the FNDDS and the ANSES/CIQUAL database. Kiwifruit had significantly higher NRF9.3 scores than all other fruit categories, except for citrus fruit and tropical fruit (FNDDS) and citrus fruit and berries (ANSES/CIQUAL). As shown by the NRF9.3 overall nutrient density metric, kiwifruit was high in other micronutrients in addition to its high content of vitamin C. Nutrient-profiling methods use fiber and multiple vitamins and minerals to identify the most nutrient-dense food items and can be applied to different databases globally. Assessing the nutrient density of fruit, with a focus on shortfall micronutrients, can help in the implementation of dietary guidance.

## 1. Introduction

Fruits are an essential component of a healthy diet [1,2]. The fruit category is a major source of under-consumed nutrients, such as vitamin C, potassium, folate, and dietary fiber [3,4]. Dietary Guidelines for Americans, 2025–2030 (DGA), views current fruit consumption as inadequate, falling short of recommended amounts [5]. DGA specifies that at least half of daily fruit intake should come from whole fruits: fresh, frozen, dried, or canned [5].

The distribution of phytonutrients does vary across fruit types. Citrus fruit and citrus juice are most strongly associated with vitamin C, bananas provide potassium and fiber, orange- and yellow-fleshed fruit such as mango and papaya are rich in provitamin A carotenoids, while berries are very high in flavonoids, notably anthocyanins [5]. While not classified as micronutrients, fruit-derived carotenoids and flavonoids have antioxidant properties and offer non-traditional health benefits [6]. The US Department of Agriculture categorizes fruits rich in those compounds as functional foods [5].

Nutrient-profiling (NP) methods are quantitative tools to assess the overall nutrient density of foods [7]. However, many existing NP models are of no help when it comes to scoring nutrient density of fruit. Neither Nutri-Score [8] nor Health Star Rating (HSR) [9] include vitamins, minerals, or phytonutrients in the calculation algorithm. Initially developed to evaluate packaged processed foods [8,9], both Nutri-Score and HSR simply penalize calories and nutrients to limit, namely, saturated fat, sugar, and sodium. While both systems award points for the fiber and fruit contents of foods [8,9], they are ill-suited to rating different types of fruit. In general, fresh fruit is low in energy and does not contain much saturated fat, added sugar, or salt [10]. Both Nutri-Score and HSR treat all fruit as equally nutrient-dense.

Past NP attempts to assess the nutrient density of fruit have rarely compared kiwifruit (*Actinidia* spp.) with other fruit or juices, even though its nutrient composition is very distinctive [11,12]. Most databases list one single kiwifruit, described as "kiwifruit, green, raw". Yet both green- and gold-fleshed cultivars are unusually rich in vitamin C, more so than citrus, and also provide dietary fiber, potassium, folate, and vitamin E [13,14]. Establishing nutrient contents requires more cultivars and a more complex evaluation tool.

The well-established Nutrient-Rich Food Index NRF9.3 goes beyond fiber and vitamin C to assess the overall nutrient density of fruit, fresh, frozen, dried, or canned [15]. The NRF9.3 is based on nine nutrients to encourage: protein, fiber, calcium, iron, potassium, magnesium, and vitamins A, C, and E [15]. Nutrients to limit are added sugar, sodium, and saturated fat [15]. Exploratory NRF models have assessed flavonoids and carotenoids in order to better capture the nutrient density of berries, spices and herbs [6]. While the NRF is an across-the-board score that applies the same nutrient standards to all food groups, it can also be applied to a single food group, in this case, fruit.

The present goal was to apply the well-established NRF9.3 metric to assess the nutrient density of kiwifruit varietals compared with other fruit and fruit juices. The goal was to show that kiwifruit presents a high-nutrient-density package. Two separate national nutrient composition databases were used, one from the US and one from France [16,17]. Additional data for kiwifruit varietals came from the New Zealand Nutrient Composition Table [18].

## 2. Methods

### 2.1. The FNDDS 2021–2023 Nutrient Composition Database

Nutrient composition data for the US came from the open-access US Department of Agriculture (USDA) Food and Nutrient Database for Dietary Studies (FNDDS) [16]. The FNDDS 2021–2023 provided energy and nutrient contents for 60 nutrients per 100 g of food, edible portion, for >5000 foods from all food groups. Additional data for added sugar were obtained from the USDA Food Patterns Equivalents (FPED) database [19].

Foods in the FNDDS database are aggregated into food categories, food subgroups and food groups. These are identified by different What We Eat in America (WWEIA) digit codes. The present sample, drawn from the USDA fruit group, included fruit, fresh, frozen, cooked, or canned, dried fruit, fruit juices, and processed fruit, including fruit cocktail, fruit salads, and fruit desserts. The FNDDS fruit group also included cooked fruit or fruit mixtures, often eaten with the addition of other ingredients (e.g., sugar and cream). Fruit juices (100% juice) were included. Fruit-based soft drinks were excluded. A total of 159 items were available for NP analysis. The WWEIA nutrient composition data are for foods as commonly consumed by NHANES participants as opposed to foods as purchased.

### 2.2. The 2025 ANSES/CIQUAL Nutrient Composition Database

Nutrient composition data for France came from the open-access ANSES/CIQUAL database. The CIQUAL (Centre d’Information sur la Qualité des Aliments) database is maintained by the French Agency for Food, Environmental and Occupational Health & Safety (ANSES Agence nationale de sécurité sanitaire de l’alimentation, de l’environnement et du travail). This is the nutrient composition database that is used in dietary surveillance studies in France, most recently in the INCA3 survey (The Third French Individual and National Food Consumption Survey). Data are available for energy and nutrient content of food per 100 g, edible portion [17]. The 2025 version lists 3484 foods, including both raw and processed foods, which are representative of the French diet. Similar to the FNDDS aggregation scheme, fruit categories were fresh, frozen, cooked, or canned, dried fruit, fruit juices, and processed fruit, including fruit cocktail, fruit salads, and fruit desserts. Included were canned fruit in sugar syrup and stewed fruit (compotes), with or without added sugar. A total of 151 items were available for nutrient-profiling analysis.

### 2.3. The New Zealand Food Composition Database

Nutrient composition data for kiwifruit came from the open-access New Zealand Food Composition Database (NZFCD), developed by the Ministry of Health and Plant & Food Research [18]. The database includes nutrient data for more than 2850 foods and ingredients commonly consumed in New Zealand. These include raw agricultural foods, notably 5 varieties of kiwifruit. Data for kiwifruit varietals (green, SunGold, and RubyRed) were used to supplement the values for green kiwifruit, already present in the US and French databases.

### 2.4. Nutrient Composition Data for Kiwifruit Varietals

There was some disagreement in the USDA data sources regarding the exact vitamin C content of kiwifruit. The Foundation database available at FoodData Central lists a single kiwifruit entry, green kiwifruit, raw (FDC ID 2710831; last updated 31 October 2024) [20], with a mean analytical vitamin C value of 58.8 mg per 100 g based on eight samples (range: 40.7–70.9 mg). The USDA Standard Reference Release (SR28), as cited in a review paper [21], provides values of 92.7 mg/100 g for green kiwifruit (Hayward) and 161.3 mg/100 g for SunGold. The value for green kiwifruit provided in the FNDDS 2021–2023 downloadable Excel file is 74.7 mg/100 g. The downloadable CIQUAL database lists the vitamin C content of green kiwifruit as 81.9 mg/100 g. The NZFCD lists the vitamin C content of green kiwifruit as 153.7 mg/100 g and that of Zespri™ SunGold kiwifruit as 152 mg/100 g [18], more than twice the FNDDS value. A cultivar available in the US, red-fleshed kiwifruit (Zespri™ RubyRed), is listed as containing 189 mg/100 g of vitamin C. Organic cultivars may differ further in their compositional profiles [21]. The FNDDS and CIQUAL databases, as currently structured, may understate the nutrient density of commercially available kiwifruit. The different values are listed in Table 1 below. The present default was to use those values that are currently listed in the three publicly available Excel databases.

### 2.5. The Nutrient-Rich Food Index NRF

The Nutrient-Rich Food Index (NRF) is a well-established nutrient-profiling model that is used to calculate overall nutrient density of foods [6,7]. The NRF index is composed of two subscores: the positive subscore NRn, based on a variable number *n* of nutrients to encourage, and the negative subscore LIM, based on 3 nutrients to limit. The NR9 subscore (original version) was based on protein, fiber, calcium, iron, potassium, magnesium, vitamin A, vitamin C, and vitamin E [15]. The value of NR9 is calculated as the sum of percent daily values (%DV) calculated per 100 kcal. The contribution of each nutrient (%DV) was capped at 100% so that no single nutrient would dominate the total score [15]. The negative subscore LIM is based on the %DV for saturated fat, added sugar, and sodium, also calculated per 100 kcal. The final NRF9.3 score is calculated as NR9 − LIM, that is, the sum of the %DV for 9 nutrients to encourage minus the sum of the %DV for the 3 nutrients to limit.

Nutrients used to construct the NRF metric include nutrients of public health concern as identified by the DGA [5], namely, fiber, potassium and calcium. Fruit does not contain much vitamin D, so vitamin E was used instead. Nutrient standards used with the US FNDDS database came from the US FDA [22]. Nutrient standards for use with the ANSES/CIQUAL database came from the French Apports Nutritionnels Conseillés [23]. The maximum recommended values (MRVs) for disqualifying nutrients were 20 g for saturated fat, 50 g for added sugar, and 2300 mg for sodium. The standards are summarized in Table 2. The algorithm for calculating NRF subscores and the total score is provided in Supplemental S1.

There were some substantial differences between the two sets of standards for nutrients to encourage, notably for potassium, calcium, vitamin C and vitamin A. In some cases, the French ANC standards were below the US values; in other cases, they were higher. For example, the French standards were higher for vitamin C (110 mg vs. 90 mg) but much lower for potassium (3500 mg vs. 4700 mg). In contrast, the MRV standards were the same in both countries.

### 2.6. Plan of Analyses

Statistical analyses were conducted to compare the nutrient profiles of kiwifruit with those of citrus fruit and juices, berries and other fruits. Comparisons were based on one-way ANOVAs, followed by inspection of 95% confidence intervals. Additional post hoc analyses used kiwifruit as the control group and Dunnett’s test to correct for multiple comparisons. While the tables and figures show more disaggregated data, statistics were conducted using aggregated food groups in order to avoid very small sample sizes.

## 3. Results

### 3.1. Analyses of FNDDS 2021–2023 Data

Table 3 summarizes the mean energy density and selected nutrient content per 100 g for selected fruit categories from the FNDDS database. The values shown are means and standard errors of the means (SEMs). The number of items in each category is also indicated.

The energy density ranged from 34.8 kcal/100 g for melons to 292.4 kcal/100 g for dried fruits. The energy density of fresh fruit was low, ranging from 34.8 kcal/100 g for melons to 76.3 kcal/100 g for other fruits. The energy density of fruit juices was in the order of 50 kcal/100 g; baked fruit preparations (185.5 kcal/100 g) and fruit salad desserts with dressing or cream (180.0 kcal/100 g) had high energy density due to added sugar and fat.

Kiwifruit (all 6 varietals combined) had the highest mean vitamin C content of any fruit category (129.3 ± 17.6 mg/100 g), more than three times that of oranges and juice (46.8 ± 11.2 mg/100 g), and nearly six times that of berries (23.4 ± 5.5 mg/100 g). Potassium was highest in dried fruits (556 ± 68 mg/100 g), followed by kiwifruit (284 ± 22 mg/100 g) and tropical fruits (317 ± 76 mg/100 g). Compared with kiwifruit, the potassium content was lower in oranges and juice (215 ± 46 mg/100 g) and in berries (128 ± 11 mg/100 g).

The dietary fiber content was highest in dried fruits (6.1 ± 0.6 g/100 g), twice that in berries (3.8 ± 0.5 g/100 g) and raw kiwifruit (2.3 ± 0.3 g/100 g). The fiber content in raw kiwifruit was lower than that in citrus fruits (3.1 ± 0.9 g/100 g) but higher than that in citrus juice (0.3 ± 0.1 g/100 g).

Most fresh fruits provide negligible amounts of vitamin E, avocado being the one exception. Both green and gold kiwifruit provide approximately 1.4–1.5 mg of α-tocopherol per 100 g [15,17], which is about 10% of the daily value. No other fresh fruit category provided a comparable amount of vitamin E.

Kiwifruit contributed relatively little vitamin A compared with orange- and yellow-fleshed fruit. Mango, apricot, and cantaloupe are substantially richer in β-carotene and β-cryptoxanthin, the principal provitamin A carotenoids in the US diet, and score higher on the vitamin A component of the NR9 subscore. In the FNDDS data, the mango and papaya category had a mean vitamin A content that exceeded kiwifruit by a substantial margin, consistent with the well-established carotenoid dominance of orange-fleshed tropical fruit. Green kiwifruit provides approximately 33 µg of dietary folate per 100 g of edible portion based on USDA analytical data (with a range of 28–38 µg for eight samples; USDA FoodData Central, 2024), exceeding the folate content in apples, pears, most stone fruit, and typical citrus entries.

### 3.2. NRF9.3 Nutrient Density Scores

**Figure 1:**
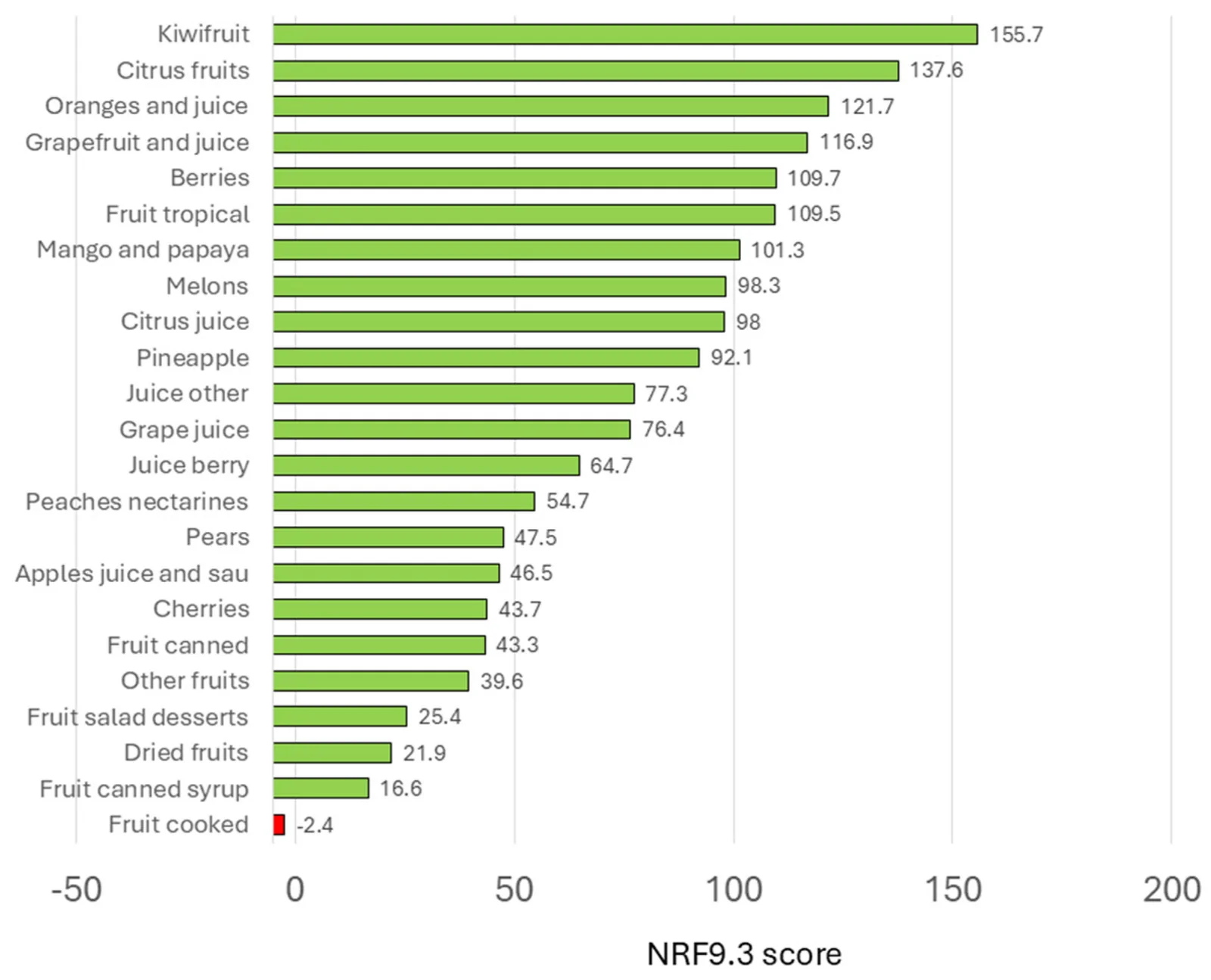
NRF9.3 nutrient density scores in descending order by fruit category. FNDDS 2021–2023 Database (*n* = 159); NRF9.3 score = NR9 − LIM per 100 kcal.

Figure 1 shows the NRF9.3 scores for fruit categories ranked in descending order. Kiwifruit had the highest mean scores (155.7), followed by citrus fruits (137.6), oranges and juice (121.7), grapefruit and juice (116.9), berries (109.7), and tropical fruit (109.5). All three kiwifruit cultivars ranked among the highest-NRF9.3-scoring fruits. The mean NRF9.3 scores for kiwifruit (*n* = 6) were higher than those for other fruit categories that have ranked highly in nutrient density analyses of fruit. Among commonly consumed fruit with high scores were mango and papaya (101.3), melons (98.3), pineapple (92.1), peaches and nectarines (54.7) and a variety of 100% fruit juices.

At the lower end of Figure 1 were pears (47.5), other fruits (29.6), fruit salad desserts with dressing (25.4), canned fruit (43.3), dried fruits (21.9), canned fruit in syrup (16.6) and cooked fruit (−2.4). Cooked fruit had the lowest NRF9.3 scores, reflecting the combined negative effects of higher energy density and added sugar or fat. Overall, the NRF9.3 scores favored fresh fruits with high concentrations of vitamin C and other qualifying nutrients per 100 kcal.

Within the fruit group, the negative LIM subscores were driven by added sugar, since most fresh and frozen fruit contain negligible amounts of saturated fat and sodium. The consequences for the NRF9.3 rank ordering were substantial: fruit salad desserts (energy density of 172 ± 16.3 kcal/100 g in FNDDS) and baked fruit preparations (227 ± 73 kcal/100 g) had some of the lowest NRF9.3 scores.

**Figure 2:**
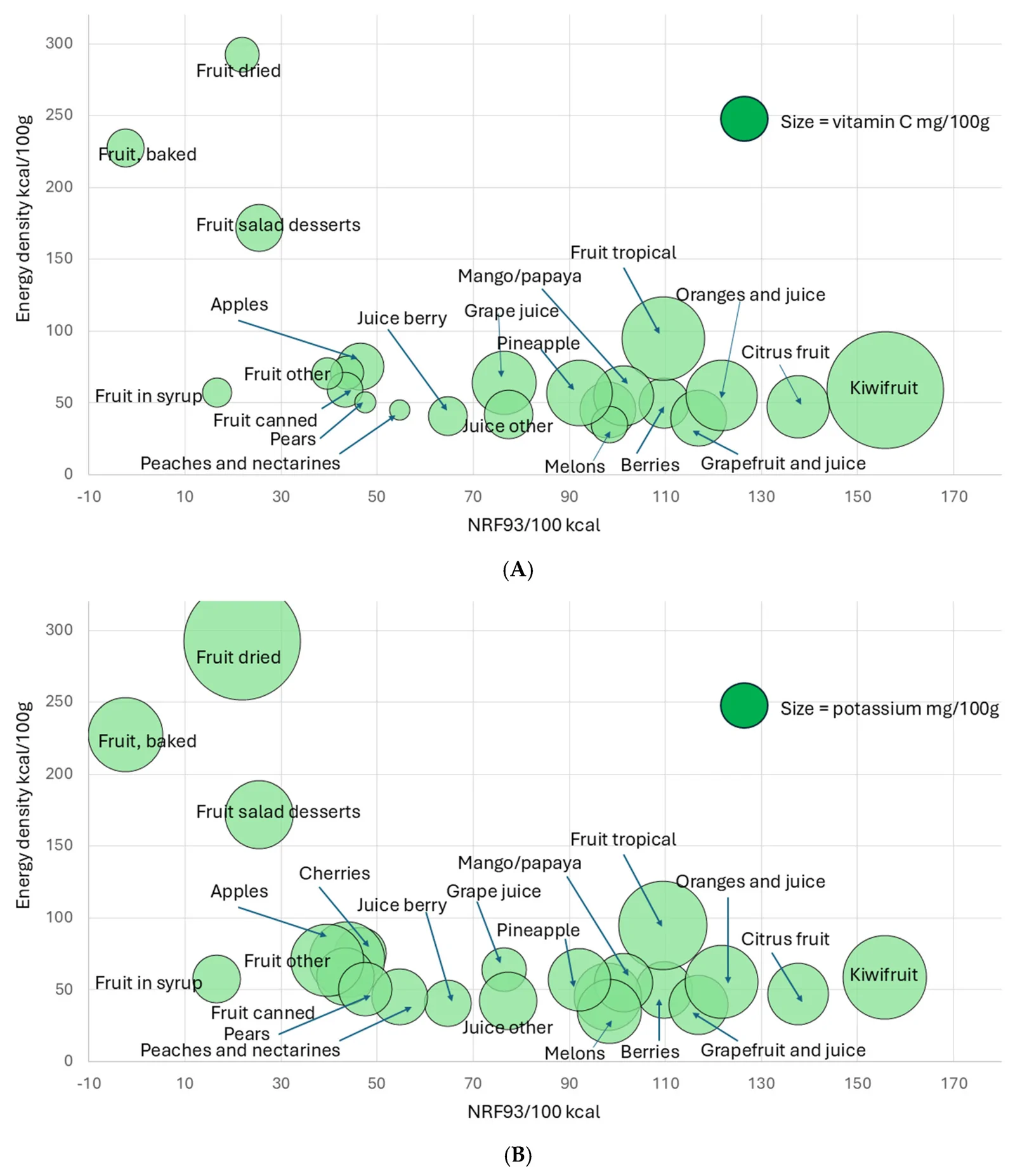
NRF9.3 scores plotted against energy density (kcal/100 g). Size of each circle is proportional to vitamin C content in mg/100 g (**A**) and potassium content in mg/100 g (**B**).

Figure 2 below shows the NRF9.3 scores plotted against energy density (kcal/100 g), with the bubble size being proportional to vitamin C content (Figure 2A) and potassium content (Figure 2B), both in mg per 100 g. The figure shows that kiwifruit had the highest NRF9.3 score, was low in energy density, and had by far the highest vitamin C content of any category.

### 3.3. The ANSES/CIQUAL Nutrient Composition Database

Table 4 shows the mean energy density and selected nutrient content per 100 g in the ANSES/CIQUAL 2025 database by fruit category. As observed in the FNDDS analysis, dried fruit was the most energy-dense (281.5 kcal/100 g; SEM: 14.3), while fresh and minimally processed fruit categories clustered between 31.8 and 86.9 kcal/100 g. Compote (stewed fruit; 83.6 kcal/100 g) and exotic fruit (86.9 kcal/100 g) were among the higher-energy-density items.

Kiwifruit had the highest vitamin C content of any fruit category, followed by currants at 105.4/100 g. The vitamin C content of citrus fruit was in the order of 46 mg/100 g, followed by mango and papaya (40.4 mg/100 g), exotic fruit (37.7 mg/100 g) and grapefruit (33.5 mg/100 g).

Potassium was highest in dried fruits (989 mg/100 g), followed by dried fig, prune, and raisin (741.6), kiwifruit (299 mg/100 g), exotic fruit (281.9 mg/100 g) and melon (236 mg/100 g). Differences between categories were statistically significant for all four nutrients (one-way ANOVA; *p* < 0.05).

The dietary fiber content was highest in dried fruits (8.9 g/100 g) and dried fig, prune, and raisin (6.2 g/100 g), followed by currants, berries, and exotic fruit. The dietary fiber content in green kiwifruit was comparable to that in oranges and other raw fruit. Melons were highest in vitamin A, and dried fruit was highest in vitamin E.

**Figure 3:**
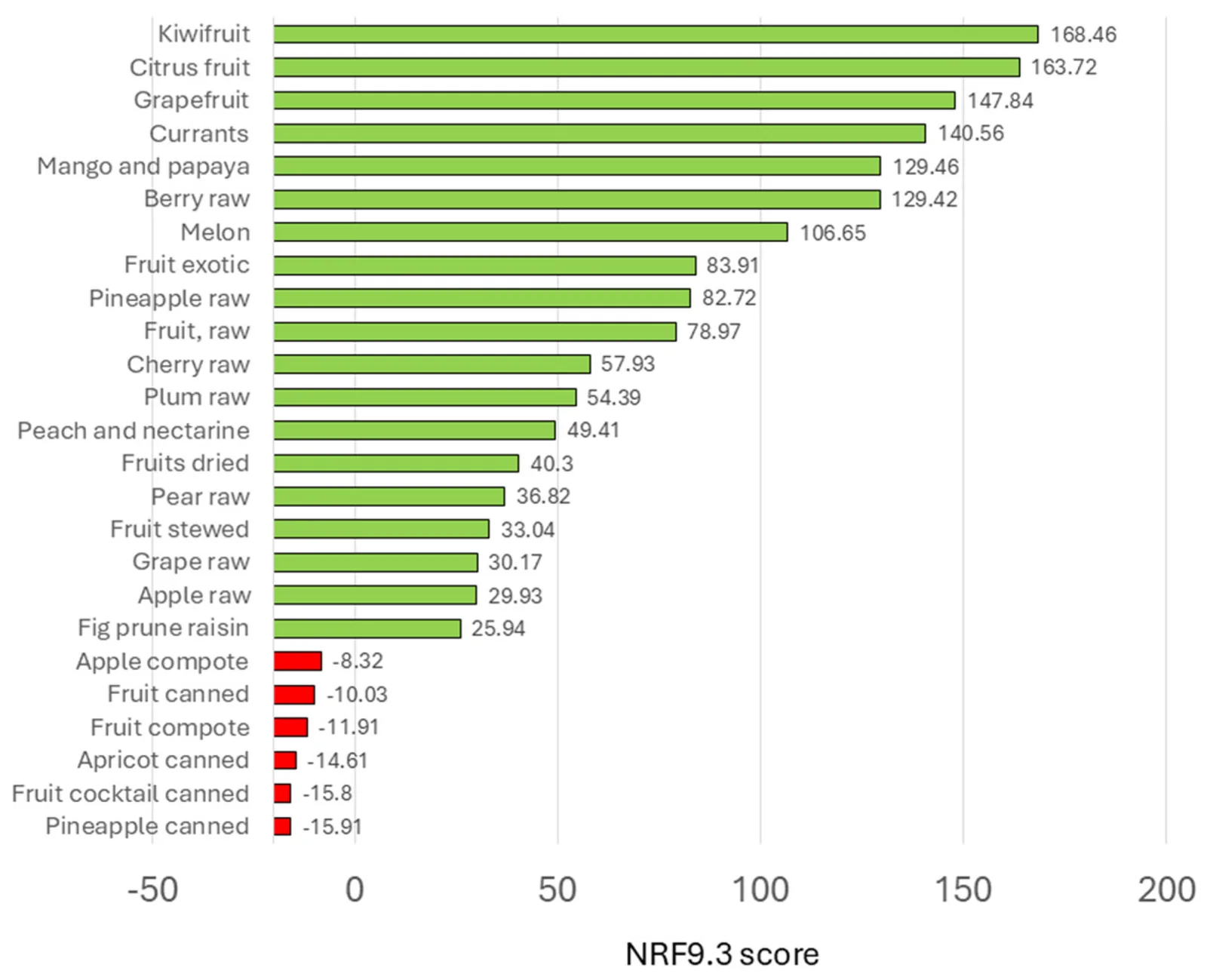
Mean NRF9.3 scores for fruit categories in the ANSES/CIQUAL 2025 database, in descending order. NRF9.3 score—NR9 − LIM.

Figure 3 shows the NRF9.3 scores for the fruit categories in the ANSES/CIQUAL 2025 database. Kiwifruit had the highest mean score (168.5), closely followed by citrus fruit (163.7), grapefruit (147.8) and currants (140.5). Mango and papaya, raw berries and melons also ranked among the highest-scoring fruit categories. At the lower end of the distribution with negative NRF9.3 scores were canned fruit in sugar syrup and compotes with added sugar, reflecting higher contributions from nutrients to limit. The overall ranking pattern was consistent with the FNDDS analysis. Fresh fruits characterized by high vitamin C contents and low energy densities occupied the upper end of the NRF9.3 distribution, whereas processed fruit products and fruit preparations containing added sugars generally had lower scores. Kiwifruit ranked among the highest-scoring fruit categories in both databases.

In the CIQUAL analysis, canned fruit in syrup, along with apple and fruit compotes, yielded negative scores, largely because of the presence of added sugar. This pattern was consistent with that observed for the FNDDS data. In the CIQUAL database, stewed fruit, compotes and canned fruit in sugar syrup were more prevalent than in the FNDDS.

**Figure 4:**
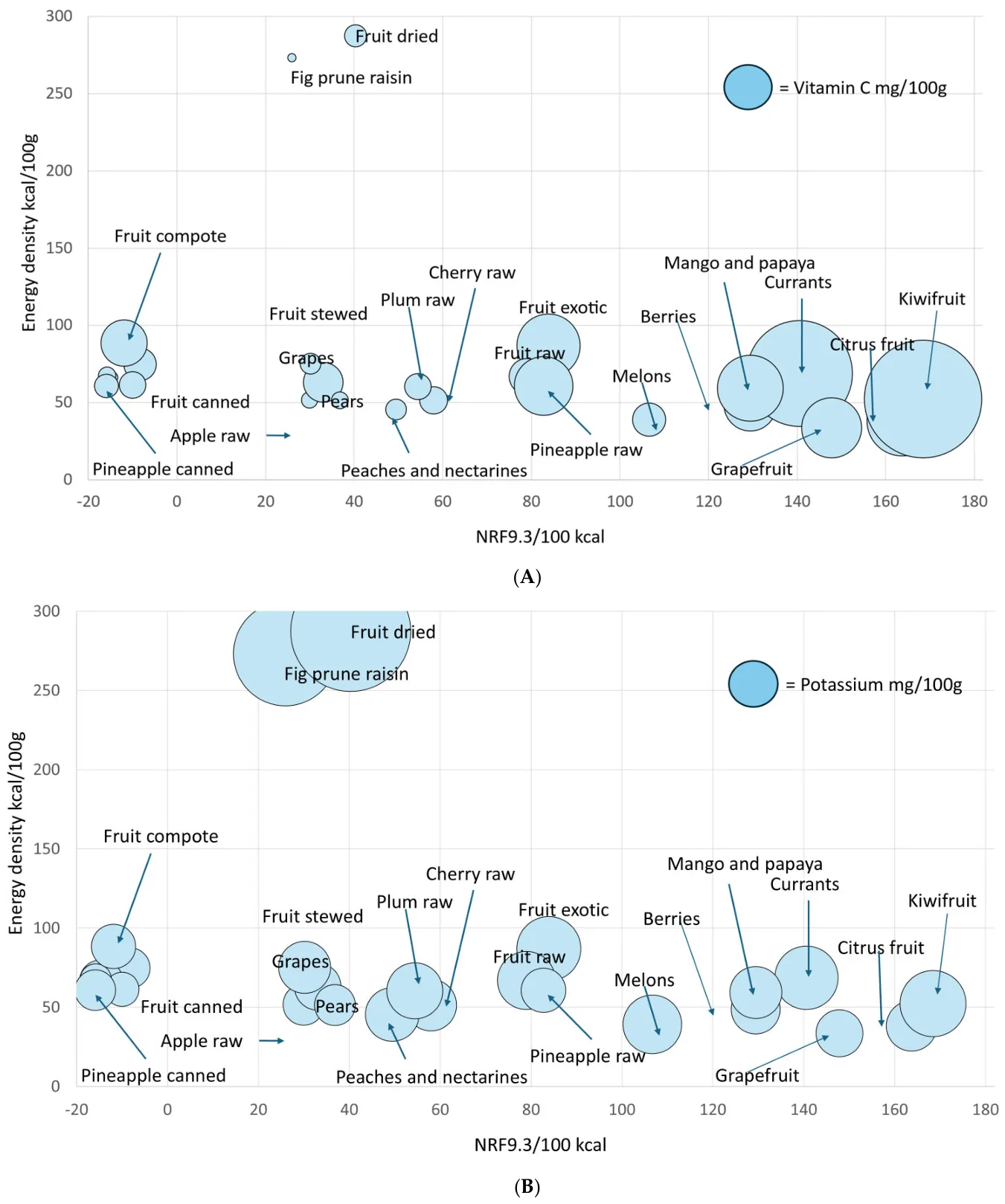
NRF9.3 scores plotted against energy density (kcal/100 g) for CIQUAL fruit categories. Size of each circle is proportional to vitamin C content in mg/100 g (**A**) and potassium content in mg/100 g (**B**).

Figure 4 shows NRF9.3 scores plotted against energy density for each CIQUAL fruit category. The size of the bubble is proportional to vitamin C content in mg/100 g (Figure 4A) and to potassium content in mg/100 g (Figure 4B). Fresh fruit and juices were of low energy density, with oranges and kiwifruit reaching the highest NRF9.3 scores.

### 3.4. Statistical Comparisons

Comparisons between kiwifruit and other aggregated fruit categories are shown in Table 5. The mean NRF9.3 scores, standard errors of the means (SEMs), and confidence intervals (CIs) are shown. One-way ANOVAs were followed by post hoc tests with kiwifruit as the control condition, using Dunnett’s test to correct for multiple comparisons. Kiwifruit (n-6) had the highest mean NRF9.3 scores in both the FNDDS and the ANSES/CIQUAL nutrient composition databases. Kiwifruit had significantly higher NRF9.3 scores than all other fruit categories, except for citrus fruit and tropical fruit (FNDDS) and citrus fruit and berries (ANSES/CIQUAL). The aggregation scheme followed the original FNDDS and CIQUAL food groups to ensure sufficient cell size.

## 4. Discussion

Kiwifruit is not only exceptionally high in vitamin C but alsocontains multiple other nutrients [13,14]. The present aim was to compare kiwifruit with other fruits and 100% fruit juices, using a well-established nutrient-profiling method, the Nutrient-Rich Food Index NRF9.3 [15]. The NRF9.3 algorithm captures multiple nutrients, including fiber, multiple vitamins and minerals, and protein. The NRF9.3 has been applied to all foods across the board [15] as well as to selected food groups. One exploratory NRF version used flavonoids to capture nutrient density of berries in relation to other fruits [6].

The present analyses used two separate national databases, one from the US and one from France. The US Department of Agriculture FNDDS is the nutrient composition database used in the What We Eat in America studies, the dietary component of the National Food and Nutrition Examination Survey (NHANES) [16]. The CIQUAL nutrient database in France is used in the nationally representative INCA dietary surveys [17]. Both databases only list a single green kiwifruit, raw. Values for additional kiwifruit varieties came from the New Zealand Food Composition Database. The vitamin C content of several Zespri cultivars, including green, Sungold and RubyRed varieties, was of particular interest [18].

Based largely on their high vitamin C content per 100 g, citrus fruit and juice have dominated nutrient density rankings in the past [6]. The NRF9.3 takes a different approach by using multiple nutrients to calculate the overall nutrient score per 100 kcal [15], rather than per 100 g. Percent daily values for each nutrient are then truncated at 100% so that a single nutrient does not have a disproportionate impact on the total score. The original version of the NRF published in 2008 used vitamin E because vitamin D data were not available at the time. Since then, category-specific NRF scores have used a variety of nutrients of concern that were provided by different food groups. In essence, the NRF is a composite nutrient-to-calorie ratio.

Nutrient-profiling methods rely on the accuracy of nutrient composition databases. One problem was that three USDA databases each provided different values for the vitamin C content of green kiwifruit. We used nutrient values provided in the FNDDS 2011–2023 Excel file, available from FoodData Central. While energy density values for kiwifruit were comparable in the FNDDS and CIQUAL databases, there were sharp differences in vitamin C and potassium contents. Given the diversity and disparity of mineral and other nutrient elements [24,25], existing databases in the US and in France (and elsewhere) may underestimate the nutrient density of kiwifruit. The publicly available NZFCD was a useful resource.

Second, NP models use established nutrient standards to calculate nutrient density per reference amount, whether 100 kcal, 100 g or serving size. As shown in Table 2, those standards can vary across countries and regions. The RDI (reference daily intake) for vitamin C was 90 mg/day in the US and 110 mg/day in France. Potassium values were 4700 mg/day in the US and 3500 mg in France. There were also substantial differences in standards for iron, calcium, magnesium and fiber. The common standard for sodium was 2300 mg/day in both countries; however, the World Health Organization uses a lower value of 2000 mg/day. As a result, nutrient density scores for the same fruit or fruit categories in the two databases were similar but not exactly the same.

Aggregating individual items into categories depends on the population food patterns. Both databases listed fresh fruit, fruit juices, and fruits that were frozen, dried, or canned. The FNDDS aggregation schemes separated citrus fruit and juices from other fruits and juices and had separate categories for berries, melons, peaches, apples and pears. Applesauce, fruit cocktails, and fruit salads with toppings were listed in the FNDDS database, whereas stewed fruits and compotes were more prevalent in France. The French database listed a number of exotic fruits (durian and dragon fruit) that were absent from the FNDDS. The French database also listed a large number of canned fruits in sugar, which were less common in the FNDDS. The limited number of fruit items (around 150) means that some categories were very small. For example, the CIQUAL database listed only one orange, whereas the FNDDS listed oranges and multiple orange juices. The aggregation schemes were determined by the respective national agencies and were largely followed here to arrive at mean NRF nutrient density values by category. The present study made an effort to keep the categories similar.

Kiwifruit held the top position for several key nutrients in both databases. Because kiwifruit’s energy density was low (58.7 kcal/100 g in FNDDS and 52.3 kcal/100 g in CIQUAL), the vitamin C percent daily value (%DV) per 100 kcal reached the 100% cap in both databases. A 100 g serving of raw kiwifruit delivered > 100% DV for vitamin C at under 60 kcal, a ratio that no other commonly consumed fresh fruit category in either the FNDDS or CIQUAL was able to achieve. The potassium content in kiwifruit ranked second among fresh fruit; vitamin E was high, which is unusual for fruit other than avocado; fiber content was comparable to that of citrus; and folate was above most temperate fruit. Melons exceeded kiwifruit in provitamin A carotenoids. In general, fruits combine low energy density with a high concentration of several micronutrients per calorie, leading to high nutrient density scores [26]. Kiwifruit, citrus fruit, and berries ranked higher on NRF9.3 than most fruit subcategories in both databases (*p* < 0.05) but were not significantly different from each other. No other fresh fruit category has achieved this across both databases.

The present NRF scoring system did not include flavonoids or carotenoids (α-carotene, β-carotene, β-cryptoxanthin, lycopene, and lutein/zeaxanthin) [6,27]. Carotenoids and flavonoids are bioactive phytochemicals, not classic micronutrients, but have antioxidant properties and non-traditional health benefits [28]. Incorporating flavonoids into the NRFa11.3 model [6,27] led to higher scores for berries, due largely to anthocyanins. Green kiwifruit contains phenolic compounds including quercetin derivatives, caffeic acid, and chlorogenic acid and has shown antioxidant activity in vitro relative to some fruits, including persimmon [28]. Zespri RubyRed kiwifruit may also have higher anthocyanin concentrations than green or gold varieties [13,14], but these data were not available in either the FNDDS or CIQUAL at the time of this study.

The NRF9.3 also includes a negative LIM subscore that is based on saturated fat, added sugar, and sodium [8]. With the exception of baked or sweetened stewed fruit, fruit salads with dressings, and canned fruit in syrup, LIM scores for the fruit category were expected to be close to zero. Dietary Guidelines for Americans, 2025–2030, recommends limiting added sugar [5]. In the context of NRF9.3, adding sugar to fruit not only increases energy density but reduces composite nutrient to calorie ratio that the NRF9.3 algorithm is designed to capture [8,29].

The NRF9.3 approach also had important limitations. As noted above, the NRF9.3 is a balanced compensatory score that is based on the sum (rather than mean) or percent daily values; other methodologies focus more heavily on the nutrients to limit. Second, the NRF score is normally calculated per 100 kcal, although alternative bases of calculation (100 g or serving size) have also been used. Third, there are no provisions for protein quality, micronutrient bioavailability, or food matrix effects. Finally, most nutrient-profiling models apply to individual foods and not to total diets.

That fruit makes an important contribution to dietary nutrient density in the US [29] and in France [30] is already well-established. Regular consumption of fruits has been linked to a reduced risk of cardiovascular diseases, cancer and obesity [1,2]. Vitamin C is regarded as a shortfall nutrient in the US [31]. Population intakes of vitamin C fell by roughly 23% between 1999 and 2018, driven largely by declining consumption of fruit juice [32]. The proportion of the population below the estimated average requirement rose from 38% to 47% during that time [32]. Adults with the lowest fruit intake, whether whole fruit or fruit juice, are more likely to fall short of both vitamin C and potassium.

Another shortfall nutrient is fiber. The DGA recommends the consumption of dried fruit, high in both potassium and fiber [33]. The DGA also recommends that at least half of daily fruit consumed should be whole fruit [5]. Although both whole fruit and 100% juice are associated with higher diet quality and higher dietary potassium and vitamin C [29,34], fiber content is higher in whole fruit. When the skin of gold-fleshed cultivars is eaten, a practice that is common in some markets and promoted by Zespri, total fiber per fruit increases above the flesh-only value.

Nutrient-profiling models rate foods based on multiple nutrients. However, those rankings can also vary depending on what standards are used and which nutrients are included. The Powerhouse Fruits and Vegetables score [35], based on 17 qualifying nutrients, placed citrus fruit and berries in the lower half of the distribution, well below leafy green vegetables. Another score was based on the antioxidant properties of selected foods, including vegetables and fruit [36]. Other NP models showed that citrus juice performed well on nutrient density measures despite its low fiber content [6]. In the same analysis, fruit salads, desserts, and other mixtures prepared with added sugar or cream scored lower and were penalized by the LIM subscore, while dried fruit, despite its high energy density, retained favorable scores on account of its fiber and potassium contents [6]. When the NRF9.3 framework was instead adapted to incorporate flavonoid content (NRF9f.3), berries rich in anthocyanins ranked among the most nutrient-dense fruit categories, comparable to citrus fruit and citrus juice. The NRF framework can be adapted, where composition data permits, to incorporate nutrients of particular relevance to fresh produce, among them folate, carotenoids, and flavonoids. Nutrient profiling can assist studies in determining the impact of fruit and vegetable consumption on human health [37].

The present analyses disaggregated fruit into separate categories, rather than treating all fruit as a single undifferentiated group, a problem for both the Health Star Rating and Nutri-Score. The NRF9.3 scores nutritional quality by summing the percent daily value (%DV), calculated per 100 kcal and capped at 100% for each nutrient, for nine nutrients to encourage, including protein, dietary fiber, vitamin C, potassium, and other essential vitamins and minerals. From this total, the model subtracts the corresponding %DVs for three nutrients to limit saturated fat, added sugar, and sodium, yielding an overall measure of nutrient density.

This study had limitations. First, NP models are bound by the quality of available data. The data for Kiwifruit came from three different national datasets. Some fruit categories were missing. Red currants and blackcurrants were not listed in the FNDDS but are popular in France. Second, the standards for calculating daily values differed between the two countries. Each NRF9.3 model was, therefore, not only category-specific but also country-specific. Third, complete data from flavonoids, polyphenols, and other phytochemicals were not available but may prove useful in the future. One strength is that the present NP methodology can be applied to different regions and different datasets.

## Figures and Tables

**Table 1:** Energy, vitamin C and potassium contents of different varietals of kiwifruit as listed in 3 national nutrient composition databases.

Kiwifruit Varietal	Database	Energy Density (kcal/100 g)	Vitamin C mg/100 g	Potassium mg/100 g
Green	FNDDS *	64	74.7	198
Green	CIQUAL **	60.9	81.9	290
Green (*n* = 3)	NZFCD ***	58.3	153.7	290
Sungold	NZFCD	54	152.0	298
RubyRed	NZFCD	59	189.0	338

* FNDDS Food and Nutrient Database for Dietary Studies, US. ** CIQUAL (Centre d’Information sur la Qualité des Aliments), France. *** NZFCD New Zealand Nutrient Composition Database, New Zealand.

**Table 2:** Reference daily values used in NRF9.3 scoring for the US (US DV) and France (ANC).

Nutrients to Encourage			Nutrients to Limit (LIM)	
Nutrient	US DV	France ANC	Nutrient	Daily Value
Protein (g)	50	50	Added sugar (g)	50
Fiber (g)	28	30	Saturated fat (g)	20
Iron (mg)	18	11	Sodium (mg)	2300 ^a^
Calcium (mg)	1300	950		
Potassium (mg)	4700	3500		
Magnesium (mg)	420	325		
Vitamin A (µg RAE)	900	700		
Vitamin C (mg)	90	110		
Vitamin E (mg α-TE)	15	12		

US DV = US Food and Drug Administration daily value. ANC = Apports Nutritionnels Conseillés (France). ^a^ The sodium maximum recommended value (MRV) used for LIM scoring is 2300 mg/d. α-TE = alpha-tocopherol equivalents; RAE = retinol activity equivalents.

**Table 3:** Energy density and selected nutrient content of fruit categories in FNDDS 2021–2023 (mean ± SEM per 100 g).

		Energy Density Kcal/100 g		Vitamin C mg		Potassium mg		Fiber g		Vitamin E mg		Folate mg		Vitamin A RAE mcg	
WWEIA Category	N *	Mean	SEM	Mean	SEM	Mean	SEM	Mean	SEM	Mean	SEM	Mean	SEM	Mean	SEM
Apples, juice, sauce	10	75.2	9.8	20.9	6.1	108.9	10.9	1.1	0.2	0.1	0.0	1.6	0.4	3.9	2.8
Berries	11	49.9	3	23.4	5.5	127.7	11.3	3.8	0.5	0.7	0.1	15.2	3.0	3.4	0.9
Cherries	2	71	0	10.4	0	230	0	2.1	0	0.1	0	4	0	3	0
Citrus fruits	5	47.2	7.9	35.9	5.3	151.6	14.6	3.1	0.9	0.2	0.0	13.6	1.7	17.2	7.3
Citrus juice	2	45	2	29	0.3	180	2	0.3	0.1	0.2	0.0	17.5	12.	7	5
Dried fruits	17	292.4	9.6	10.9	3.37	556.3	67.9	6.1	0.6	1.1	0.3	14.2	4.4	43.9	14
Fruit canned	18	59	3.4	11.3	3.0	131.9	8.4	1.4	0.1	0.3	0.1	7.8	1.9	20.8	5.6
Fruit canned syrup	6	57.3	1.7	7.7	5.0	90.8	11.5	1.1	0.1	0.2	0.1	3	0.7	13.3	6.8
Fruit cooked	5	227.4	73.1	12.9	8.6	222.4	96.7	3.3	1.2	0.3	0.2	6.6	2.7	7.4	5.2
Fruit salad desserts	13	171.9	15.9	20.9	3.6	186.5	7.1	1.7	0.1	0.4	0.1	17.9	0.8	10.5	3.8
Fruit tropical	6	94.8	30.1	64.6	34.0	317.2	76.4	4.5	1.4	0.2	0.1	19	6.0	17.5	10.4
Grape juice	2	64	2	37.7	12.7	77	27	0.2	0	0	0	1	1	0	0
Grapefruit and juice	6	39.3	0.8	29.5	2.6	138.2	4.8	0.5	0.2	0.2	0.0	12.7	1.2	13.2	9.3
Juice berry	5	40.6	2.6	13.9	5.6	85.6	13.5	0.4	0.3	0.6	0.2	8.6	2.8	2.2	1.0
Juice other	8	42.1	6.4	22	5.0	134	33.7	0.4	0.2	0.2	0.1	3.4	1.2	12.7	6.1
Kiwifruit	6	58.7	2.7	129.3	17.6	284	22.0	2.3	0.3	1.4	0.1	53.7	9.7	5.8	1.1
Mango and papaya	4	55	4.1	34.2	11.9	137.3	35.6	1.4	0.3	0.6	0.2	31.3	9.8	43.3	8.6
Melons	4	34.8	1.7	12.3	2.1	165.8	23.9	0.7	0.1	0	0.0	12	3.3	87.8	51.3
Oranges and juice	10	55.3	10.4	46.8	11.2	215.3	46.4	0.6	0.2	0.2	0.0	27.6	6.3	12.5	5.1
Other fruits	10	70.5	4.9	8.9	1.6	213.7	19.5	2.1	0.3	0.4	0.1	10.5	3.3	23.1	11.1
Peaches nectarines	3	45	1	3.7	0.4	125	3	1.5	0	0.7	0.0	6	0	23	1
Pears	2	50.5	8.5	4.1	0.2	112.5	8.5	3.4	0.2	0.1	0	7.5	0.5	0.5	0.5
Pineapple	4	56.8	1.9	40.3	13.8	154.5	19.9	0.5	0.2	0.1	0.1	19.5	1.5	1.5	0.9
Total	159	97	7.1	25.6	2.76	206.3	13.9	2.2	0.2	0.4	0.05	13.9	1.2	17.9	2.6

* N = number of food items within each WWEIA category.

**Table 4:** Energy density and selected nutrient content per 100 g of fruit categories in the ANSES/CIQUAL 2025 database (mean ± SEM).

CIQUAL Category	N	Energy Density Kcal/100 g		Vitamin C mg		Potassium mg		Vitamin E mg		Vitamin A RAE mcg	
		Mean	SEM	Mean	SEM	Mean	SEM	Mean	SEM	Mean	SEM
Apple compote	5	74.8	10.8	9.6	2.0	114.2	4.3	0.2	0.1	0.9	0.3
Apple raw	10	51.7	0.9	2.3	0.4	116.3	5.3	0.1	0.0	2.9	0.6
Apricot canned	5	66.2	1.8	1.2	0.4	130.4	4.5	0.6	0.0	29.8	14.1
Berry raw	11	48.8	1.6	26	7.1	165.8	20.5	0.9	0.1	4.5	1.4
Cherry raw	2	51.4	2.3	7	3.0	181.5	8.5	0.1	0.0	42.2	22.0
Citrus fruit	6	38.3	3.0	46	4.2	175.3	12.3	0.2	0.1	2.3	2.0
Currants	2	68.7	1.5	105.4	75.6	276	46.0	1.2	0.0	5.2	3.1
Fig prune raisin	5	273.2	15.4	0.6	0.2	741.6	59.7	0.7	0.3	10.7	6.0
Fruit canned	6	61.3	0.8	6.5	3.8	79.6	7.0	0.3	0.1	6.8	3.5
Fruit cocktail canned	4	67.5	3.5	2.7	1.2	68.4	2.5			10.5	1.3
Fruit compote	8	88.5	6.7	20.2	5.1	134.5	6.6	0.5	0.1	8.6	1.4
Fruit exotic	13	86.9	15.8	37.7	16.9	281.9	44.8	0.3	0.1	12.1	7.0
Fruit stewed	12	63	5.4	14.8	2.3	150.1	28.0	0.3	0.1	6.6	2.7
Fruit, raw	12	66.9	8.2	11.5	2.5	230.2	20.6	0.3	0.1	27.3	18.9
Fruits dried	7	287.4	22.6	4.3	1.8	989	215.6	2.7	0.9	11.2	8.2
Grapes raw	5	75.3	3.2	4	1.2	185	10.7	0.5	0.1	2.3	0.9
Grapefruit	3	33.5	0.0	33.6	1.5	151.3	10.5	0.2	0.1	29.2	16.2
Kiwifruit	6	52.3	2.6	130.5	16.9	299.3	13.8	1.3	0.1	3.2	0.5
Mango and papaya	4	59.4	12.4	40.3	15.9	185	21.8	1.1	0.5	93.1	29.2
Melon	3	39.1	11.0	10.1	4.1	236	80.9	0.1	0.0	104.2	59.3
Peach and nectarine	8	45.5	1.4	3.9	0.4	198.1	6.8	0.5	0.1	6.5	2.0
Pear raw	4	51.4	1.8	2.6	0.6	115.3	7.0	0.1	0.0	1	0.3
Pineapple canned	4	60.8	3.1	5.1	0.1	113.3	8.9	0	0.0	1.5	0.2
Pineapple raw	2	60.7	9.1	32.2	13.9	135	5.0	0.1	0.0	3.7	1.9
Plum raw	4	60.4	6.8	6.3	1.6	215.7	29.5	1	0.4	26.8	5.9
Total	151	79.2	5.4	21.1	3.0	238.4	21.1	0.6	0.1	14.9	2.9

**Table 5:** Comparing NRF9.3 nutrient density scores for kiwifruit relative to other fruit categories. N is the number of items per category. Databases are the Food and Nutrient Database for Dietary Studies (FNDDS) and ANSES/CIQUAL.

	FNDDS (*n* = 159)							ANSES/CIQUAL Database (*n* = 150)					
	N	Mean	SEM *	95% CI **				N	Mean	SEM	95% CI		
				Lower Bound	Upper Bound	*p*					Lower Bound	Upper Bound	*p*
Berries	11	109.68	13.14	80.4	138.95	0.059	Apple raw	10	29.93	1.23	27.14	32.71	<0.001
Citrus fruit	7	126.3	14.16	91.65	160.96	0.509	Berry raw	11	129.42	15.59	94.67	164.16	0.284
Dried fruits	17	21.91	4.41	12.56	31.26	<0.001	Citrus fruit	8	155.88	9.4	133.66	178.1	0.998
Fruit canned	24	36.65	6.96	22.25	51.06	<0.001	Dried fruit	13	45.43	11.93	19.43	71.43	<0.001
Fruit cooked	11	19.51	12.58	−8.52	47.53	<0.001	Fruit canned	19	−13.69	2.21	−18.33	−9.05	<0.001
Fruit desserts	13	25.43	6.22	11.87	38.98	<0.001	Fruit compote	16	−10.15	3.67	−17.99	−2.32	<0.001
Fruit tropical	6	109.49	22.18	52.48	166.49	0.121	Fruit exotic	13	83.91	15.64	49.84	117.98	<0.001
Fruits orange	10	93.98	11.3	68.42	119.55	0.005	Fruits orange	15	82.2	12.86	54.61	109.79	<0.001
Grapefruit	6	116.89	5.08	103.83	129.94	0.258	Fruit other	6	71.4	25.12	6.84	135.97	<0.001
Juice other	21	69.3	8.62	51.31	87.28	<0.001	Fruit stewed	9	46.89	18.59	4.03	89.76	<0.001
Oranges and juice	10	121.65	7.03	105.75	137.55	0.276	Fruit raw	19	73.27	11.1	49.95	96.58	<0.001
Fruit other	17	49.84	9.47	29.76	69.91	<0.001	Grape raw	5	30.17	2.65	22.82	37.52	<0.001
Kiwifruit	6	155.66	3.55	146.53	164.79	Control	Kiwifruit	6	168.46	7.78	148.47	188.45	Control
Total	159	66.91	4.16	58.7	75.12		Total	150	59.11	5.33	48.57	69.64	

* SEM—standard error of the mean; ** CI—confidence interval; *p*-value based on one-way ANOVA with post hoc tests using Dunnett’s correction for multiple comparisons. Dunnett *t*-tests treat one group as a control and compare all other groups against it. The mean difference is significant at the 0.05 level.

## Data Availability

The FNDDS and CIQUAL databases are available online. The FNDDS 2021–2023 database can be downloaded from https://www.ars.usda.gov/northeast-area/beltsville-md-bhnrc/beltsville-human-nutrition-research-center/food-surveys-research-group/docs/fndds-download-databases (accessed on 12 July 2026). The ANSES/CIQUAL 2025 French food composition table can be downloaded in Excel format from https://ciqual.anses.fr/#/cms/download/node/20 (accessed on 12 July 2026). The New Zealand Food Composition Data: FOODfiles 2024 is also available online: https://www.foodcomposition.co.nz/ (accessed on 12 July 2026). Further information can be obtained from the authors.

## Supplementary Materials

The following supporting information can be downloaded at https://www.mdpi.com/article/10.3390/foods15183232/s1, Supplemental S1. Drewnowski and Mgaya.
