# Supplementary material for: Nutrient Rich Foods Index NRF9.3 Rates Nutrient Density of Kiwifruit in the US and in France
Source: Foods. 2026 Sep 12;15(18):3232. doi: 10.3390/foods15183232 (PMC13606528; doi:10.3390/foods15183232)
Supplement: Supplementary file 1 [file foods-15-03232-s001.zip › foods-4511273-supplementary.pdf]

Supplemental S1. Drewnowski and Mgaya.

Several versions of the NRF index are available; some were developed specifically for different food groups and are therefore category specific. The NRF9.3 (standard version) measures nine nutrients to encourage: protein, fiber, calcium, iron, potassium, magnesium, vitamin A, vitamin C, and vitamin D. However, the positive NRn sub score can use different number n of nutrients to encourage. The negative LIM sub score is based on the same 3 nutrients to liit: saturated fat, added sugar and sodium.

The final NRFn.3 scores (Equation (S3)) are calculated as the sum of percent daily values (DV_n) for n nutrients to encourage (Nut_inc_i) minus the sum of percent daily values (maximum recommended value, MRV_j) for the three nutrients to limit (Nut_lim_3). Percent daily values are normally calculated per 100 kcal of food and are capped at 100%. Some versions of the NRF have calculated percent daily values per 100 g or per serving. The final NRFn.3 score, based on percent daily values, is therefore continuous rather than point based.

The NRFn.3 nutrient standards for the FNDDS data base were taken from the Food and Drug Administration. Nutrient standards for the ANSES/CIQUAL database were taken from the French Apports Nutritionnels Conseilles. Otherwise, the default nutrient standards can come from the Codex Alimentarius. The mathematical expressions are as follows:

$$\mathrm{NR}n = \left(\frac{\mathrm{Nut\_inc_1}}{\mathrm{DV_1}} + \frac{\mathrm{Nut\_inc_2}}{\mathrm{DV_2}} + \ldots + \frac{\mathrm{Nut\_inc}_i}{\mathrm{DV}_n}\right) \times (100/\mathrm{ED}) \quad \text{(S1)}$$

$$\mathrm{LIM} = \left(\frac{\mathrm{Nut\_lim_1}}{\mathrm{MRV_1}} + \ldots + \frac{\mathrm{Nut\_lim_3}}{\mathrm{MRV_3}}\right) \times (100/\mathrm{ED}) \quad \text{(S2)}$$

$$\mathrm{NRF}n.3 = \left[\sum_{i=1}^{n} \frac{\mathrm{Nut\_inc}_i}{(\mathrm{DV}_i)} - \sum_{j=1}^{3} \frac{\mathrm{Nut\_lim_3}}{(\mathrm{MRV}_j)}\right] \times (100/\mathrm{ED}) \quad \text{(S3)}$$
